# Determinants of better health: a cross-sectional assessment of positive deviants among women in West Bengal

**DOI:** 10.1186/1471-2458-13-372

**Published:** 2013-04-20

**Authors:** Katelyn NG Long, Lisa H Gren, Chris A Rees, Joshua H West, Parley Cougar Hall, Bobbi Gray, Benjamin T Crookston

**Affiliations:** 1 Department of Family and Preventive Medicine, University of Utah, Salt Lake City, UT, USA; 2 Department of Health Science, Brigham Young University, Provo, UT, USA; 3 Freedom from Hunger, Davis, CA, USA

**Keywords:** India, Women, West Bengal, Positive deviance, Health, Education

## Abstract

**Background:**

Rural women in West Bengal have been found to have low rates of formal education, poor health knowledge, high rates of malnutrition and anemia, and low levels of empowerment. Despite these difficult circumstances, some women have positive health outcomes compared to women with similarly disadvantaged backgrounds. The purpose of this study is to identify factors associated with positive health outcomes among women with primary education or less.

**Methods:**

Multivariable regression models were built for outcomes of positive deviance to better characterize the factors in a woman’s life that most impact her ability to deviate from the status quo.

**Results:**

Positive deviants in this context are shown to be women who are able to earn an income, who have access to information through media sources, and who, despite little schooling, have marginally higher levels of formal education that lead to improved health outcomes.

**Conclusions:**

Study findings indicate that positive deviant women in disadvantaged circumstances can achieve positive outcomes amidst a host of contextual barriers that would predict poor health outcomes. Focusing on areas such as enhancing access to media sources, facilitating self-help groups for married women, and promoting prolonged education and delayed marriage for girls may improve health knowledge and behavior among married women with low levels of education.

## Background

Women in West Bengal, India have been shown to have inadequate knowledge regarding HIV/AIDS [1], female reproductive issues [2], and communicable disease [3]. Research focused on mothers in West Bengal has shown low rates of oral contraceptive use [4,5], high rates of unwanted sexual encounters [6], and high rates of cervical cancer [7]. Moreover, women in West Bengal experience food insecurity [8], which has been associated with malnutrition [9,10] and anemia [11]. Low education [12], poverty and lack of empowerment [13], all of which are common in West Bengal, are among the most common risk factors for poor health behaviors. These challenges facing mothers in West Bengal have given rise to programs designed to reduce negative impacts, and include microfinance groups, education groups, and self-help groups (SHGs). Microfinance groups (including credit, savings, insurance and other financial services for the poor) have been shown to decrease poverty and health access inequities [14], increase the ability of members to seek formal health care [15], lead to improved nutritional status among participants [16], and improve social status through further work opportunities [17,18]. SHGs have been shown to decrease emotional stress [19], reduce infant mortality rates through preventive education [20], and increase HIV/AIDS knowledge [21]. Despite these favorable outcomes, SHGs and microfinance groups have been criticized for cultural insensitivity [22] and a failure to improve some basic health practices, such as contraceptive use [23].

Positive deviance is a phenomenon often found in resource-poor communities “where a few individuals and families employ uncommon, beneficial practices that allow them and their children to have better health as compared to their similarly impoverished neighbors” [24]. Positive deviance is founded on the idea that the most appropriate solutions to challenges are not found externally, but rather already exist within a given population [24]. This relies upon a community’s existing resources and strengths to achieve sustainable and culturally appropriate improvements to health. Positive deviance involves identifying unique individuals, or positive deviants, within a community who engage in uncommon practices enabling them to achieve better results than others in their community with similar resources. Once identified, positive deviants can share solutions to mutual challenges with other community members. Programs focused on identifying, understanding, and utilizing positive deviants have been shown to improve nutritional status [25,26], obesity [27], pregnancy outcomes [28], HIV/AIDS prevention behavior [29], psychosocial outcomes such as more responsive parenting, and breastfeeding [30].

The purpose of this study was to examine behaviors and attitudes exhibited by positive deviants among a sample of women in West Bengal who are married, have children, are involved in a SHG, and have little or no formal education. Specifically, this study sought to identify factors associated with positive health outcomes among women with primary education or less. Identifying these factors can assist a variety of organizations in selecting strategies to improve health knowledge, behaviors, and outcomes more appropriately among disadvantaged populations.

## Methods

### Population

Data for this study came from a community-based, cluster-randomized controlled trial conducted in a rural population in the Nadia district of West Bengal, India from 2006 to 2009 [31,32]. The study was carried out by Freedom from Hunger, an international organization that focuses on integrated microfinance, health education and social services, and Reach India, a social enterprise that provides training to local organizations that facilitates womens’ self-help groups (SHGs). Reach India trained a local NGO, Sri Mayapur Vikas Sangha (SMVS), to provide non-formal education on health and finance to women and their adolescent daughters and daughters-in-law through the SHGs. Surveys were completed for a total of 2227 consenting women and adolescent girls. An additional 81 potential respondents were approached, but declined to participate resulting in an overall response rate of 96.5%. Of the 2227 women and girls who completed surveys, 537 were married, had children, were members of a SHG, and had primary education or less. These 537 women comprised the study population for our investigation of positive deviants.

### Measurement

Five outcomes of importance to women in resource-poor settings were identified and analyzed to see which factors may have affected a woman’s ability to make healthier, more informed choices despite having low levels of education. The five outcomes were: 1) belief in a later age for marriage; 2) belief that a girl should stay in school longer; 3) awareness of HIV/AIDS; 4) correct knowledge of menstruation; and 5) perceived ability to travel alone to another village for health services. Each of these outcomes has been well established in the literature as a protective factor for women in resource-poor settings. Early marriage for girls and women is a predictor for a myriad of undesirable health and social outcomes including increased poverty, gender inequality, decreased education, HIV infection, cervical cancer, and child mortality [33-37]. Likewise, access to education is widely considered both a structural and social determinant of health that holds great promise for women in resource-poor settings [35]. Increasing women’s awareness of HIV/AIDS is one of several key objectives in preventing this epidemic and has been a United Nations’ Millennium Development Goal indicator for combating HIV/AIDS [38]. There remains much to do to achieve this goal, as up to 40% of some female populations in India have never heard of HIV/AIDS [39,40]. Women who lack knowledge about menstruation are vulnerable to a variety of sexual health challenges including reproductive tract infections and psycho-social stress related to menarche [41-43]. Finally, perceived ability to travel alone to another village for health services is a measure of empowerment that has been identified as key to womens’ health [44].

A number of variables in the original dataset were combined to create composite variables for this analysis. A food security score was created using questions from a modified version of the US Household Food Security Scale Module (US HFFSM) [45-47]. Several questions on health knowledge were included in the questionnaire. We used correct responses to “The time during the month when a female loses blood happens because_______?”, “Have you heard about HIV/AIDS?”, and “If a child has diarrhea, would you give him or her ‘more’, ‘less’, ‘the same’, or ‘nothing’, to drink?” to create a variable that indicated 0 through 3 correct answers. For analyses where having the correct answer was the outcome of interest, we removed that question from the composite and modeled health knowledge on 0 to 2 correct answers for the remaining questions. We created a variable “earnings” that included money received as a gift as well as money earned, which was subsequently put into savings. This variable was analyzed in quartiles.

### Ethical considerations

RTI International’s Committee for the Protection of Human Subjects approved this study. There were no adverse events either observed or reported during this study.

### Survey development

Several existing survey instruments were adapted for use in this study, including the Demographic and Health Survey (DHS) and a youth survey developed by the International Center for Research on Women (ICRW) for their Development Initiative on Supporting Healthy Adolescents (DISHA) program [31]. Questions included information on demographics (age, education, religion, household composition, food security and socio-economic indicators), general health behavior and knowledge indicators (HIV, diarrhea, disease prevention, etc.), and indicators of empowerment.

### Survey administration

Freedom from Hunger, Reach India, and the Centre for Micro Finance trained a team of local female investigators to administer the surveys. Prior to implementation, the survey was pilot tested and female interviewers were trained on specific strategies for interviewing women and their adolescent daughters and daughters-in-law. Data used in this analysis were collected in May and June of 2008, prior to the rollout of the nonformal health and financial education.

### Data analysis

Data were double entered, with any discrepancies being reconciled with hard copies of survey questionnaires. Statistical analyses were conducted using Stata® statistical software (version 11.1, College Station, TX, USA). Multivariable regression models were built for each of the five outcomes of positive deviance to better characterize the factors in a woman’s life that most impacted her ability to deviate from the status quo. Core linear models were built for continuous outcomes for age in years for 1) belief in a later age for a girl to get married, and 2) belief in a later age for a girl to complete her studies. We also examined a logistic model evaluating belief in later marriage age (>=18 years relative to <18 years). Core logistic models were built for 3) awareness of HIV/AIDS, 4) understanding the reason for menstruation, and 5) empowerment (traveling to another village for health services) (Table 1). All multivariable regression analyses adjusted for age, highest grade completed, and food security. Three of the models (belief in a later age for a girl to get married; HIV/AIDS awareness; and correct understanding of menstruation) were also adjusted for religion due to its significant role in improving the model’s explanatory power. Several independent variables were identified by the investigators as potential predictors of positive deviance including number of pregnancies; age at first pregnancy; hand washing behavior; who women talked to about health and reproductive issues; number of rooms in their dwelling; and items within their household like television, radios, and cameras. These variables were added individually to the linear and logistic models and were analyzed for significance within the model (p < 0.05) and significant improvement of the models’ explanatory power (increase of Pseudo R^2^ or Adjusted R^2^). Regression diagnostics were run for all models including tests for normality of continuous variables and multicollinearity among independent variables.

**Table 1:** Characteristics of outcome variables in the 5 core models

**Outcome variable (Linear)**	**Answer scale**	**Mean (sd)**	**Median**	**Range**
What is the ideal age for a girl to get married?	Single year of age	18.71 (1.53)	18	10-26
What is the ideal age a girl should finish her studies?	Single year of age	18.55 (2.70)	18	10-30
**Outcome variable (Logistic)**	**Answer scale**	**n (%)**		
Have you heard about HIV/AIDS?	Yes	146 (27.19)		
	No	391 (72.81)		
The time during the month when a female loses blood happens because?	**Correct:**			
	An egg has not united with a male sperm	166 (30.91)		
	**Incorrect:**			
	A woman is not eating enough	8 (1.49)		
	A woman is not clean	22 (4.10)		
	Do not know	330 (61.45)		
	Other	11 (2.05)		
Can you go to a health service in a nearby village unescorted or alone?	Yes	334 (62.20)		
	No	203 (37.80)		

## Results

Demographic characteristics for the participants are provided in Table 2. Results from the linear regression model predicting belief in a later age for a girl to finish her studies are provided in Table 3. A one-year increase in ideal age of marriage predicted a 0.3-year increase in the ideal age for a girl to finish her studies (β=0.301, p < 0.001, [95% CI: 0.15, 0.45]). Women that reported greater health knowledge also thought the ideal age to finish school was nearly 1 year older compared to women who responded incorrectly to a series of health related questions (β=0.871, p < 0.05, [95% CI: 0.23, 1.51]). A similar logistic model examining belief in a later age for a girl to get married found that compared to women who felt that a girl should finish her studies at a younger age (before age 18), women who believed in an older age for finishing studies had 3.8 times higher odds of thinking that the ideal age for marriage was age 18 or older (p = 0.001, [95%CI: 1.68, 8.68]) (Data table not included).

**Table 2:** **Demographic characteristics of 537 women meeting study criteria **^**a**^

	**Mean (sd)**	**Median**	**Range**	**% Yes**
Age (years)	32.42 (8.97)	31	16-63	
Attended school				47.86
Highest grade passed	3.82 (1.50)	4	1-6	
Age at marriage	15.65 (2.26)	15	10-30	
Age at first pregnancy	17.33 (2.65)	17	12-32	
Number of times pregnant	3.12 (1.49)	3	1-11	
Number of children	2.66 (1.19)	2	1-8	
Earned money in the last 6 mo.				45.23
Rupees Earned in last month	183.90 (357.74)	0.00	0-3000	
Food Secure				53.45
Number of rooms in dwelling	1.76 (0.85)	2	0-7	
Religion				
Hindu				49.35
Muslim				50.65

^a^ Study criteria: married, having one or more children, and having primary education or less.

**Table 3:** Linear regression model for ideal age for a girl to finish her studies

**Independent variables**	**Unadjusted**			**Adjusted**		
	**β Coefficient**	**CI 95%**	***P*-value**	**β Coefficient**	**CI 95%**	***P*-value**
Age^a^	0.01	−0.02, 0.04	0.387	0.02	−0.002, 0.05	0.066
Highest grade completed^b^	0.19	0.09, 0.29	**<0.001**	0.17	0.06, 0.28	**0.002**
Food Security (ref: food secure)	0.03	−0.44, 0.49	0.905	0.11	−0.34, 0.57	0.632
Ideal age for a girl to get Married^c^	0.32	0.17, 0.47	**<0.001**	0.30	0.15, 0.45	**<0.001**
Health Knowledge^d^ (ref: 0 correct responses)						
1 correct response	−0.02	−0.57, 0.53	0.949	- 0.19	−0.74, 0.36	0.496
2-3 correct responses	1.19	0.56, 1.81	**<0.001**	0.87	0.23, 1.51	**0.008**

Model included 524 respondents; r^2^ = 8.68%; constant = 11.62 years.

^a^age: 16–63 years old.

^b^highest grade finished: 0–6.

^c^ideal age for a girl to get married: 10–26 years old.

^d^Health Knowledge scale composed of 3 questions: “Have you ever heard of HIV/AIDS?”, “The time during the month when a female loses blood happens because____?”, “If a child has diarrhea, would you give him or her ‘more’, ‘less’, ‘the same’, or ‘nothing’, to drink?”.

Logistic regression modeling awareness of HIV/AIDS (Table 4) demonstrated that women who correctly answered questions about menstruation and diarrhea treatment had 2.7 times the odds of knowing about HIV/AIDS compared to women who answered neither question correctly (p < 0.05, [95%CI: 1.29, 5.46]). Women who reported receiving health information from the television or radio had 7.7 times the odds of knowing about HIV/AIDS than women who only received health information from friends and family (p < 0.001, [95%CI: 4.38, 13.60]). In order to avoid over-fitting the model, other independent variables of interest were evaluated in separate models comprised of the HIV/AIDS core model variables plus one additional independent variable. These models are presented in Table 5.

**Table 4:** Logistic regression model for knowledge of HIV/AIDS

**Independent variables**	**Unadjusted**			**Adjusted**		
	**OR**	**CI 95%**	***P*-value**	**OR**	**CI 95%**	***P*-value**
Age (ref: <25 years old)						
25-34	1.51	0.91, 2.50	0.111	1.90	1.04, 3.46	**0.037**
35+	0.77	0.45, 1.30	0.324	1.00	0.53, 1.89	0.992
Highest grade completed (ref: no schooling)						
1-3 grade	1.86	1.08, 3.21	**0.025**	1.50	0.81, 2.76	0.198
4-6 grade	3.93	2.52, 6.14	**<0.001**	2.55	1.50, 4.33	**0.001**
Food security (ref: food secure)	0.89	0.61, 1.31	0.564	0.88	0.56, 1.38	0.567
Religion^a^ (ref: Hindu)	0.57	0.39, 0.83	**0.004**	0.84	0.53, 1.33	0.466
Health knowledge^b^ (ref: 0 correct responses)						
1 correct response	1.42	0.91, 2.22	0.120	1.18	0.70, 1.99	0.532
2 correct responses	3.69	2.01, 6.74	**<0.001**	2.66	1.29, 5.46	**0.008**
TV/Radio as source of health information^c^ (ref: family & friends only)	9.75	5.70, 16.67	**<0.001**	7.72	4.38, 13.60	**<0.001**

Model included 526 respondents; pseudo r^2^ = 18.20%.

^a^ Religion: Hindu vs. Muslim.

^b^ Health Knowledge scale composed of 2 questions: “The time during the month when a female loses blood happens because____?” and “If a child has diarrhea, would you give him or her ‘more’, ‘less’, ‘the same’, or ‘nothing’, to drink?”.

^c^ Women were asked to identify sources of health information in addition to family and friends.

**Table 5:** **Logistic regression model for knowledge of HIV/AIDS (core variables + single independent variables)**^**a**^

**Independent variables**	**Unadjusted**			**Adjusted for core variables**		
	**OR**	**CI 95%**	***P*-value**	**OR**	**CI 95%**	***P*-value**
Time in a self-help group (ref: less than one year)	1.52	1.02, 2.27	**0.041**	1.58	1.03, 2.44	**0.037**
Savings (ref: 0–219 Rupees)						
220 – 719 rupees	1.54	0.84, 2.80	0.160	1.59	0.84, 3.00	0.156
720 – 1439 rupees	1.84	1.02, 3.32	**0.041**	1.86	1.00, 3.48	**0.051**
> 1440 rupees	2.63	1.50, 4.63	**0.001**	3.43	1.85, 6.36	**<0.001**
Ideal age for a girl to finish her studies (ref: <age 18)						
18-20 years old	2.05	1.28, 3.29	**0.003**	2.01	1.23, 3.29	**0.006**
21+ years old	3.61	2.04, 6.38	**<0.001**	3.17	1.73, 5.80	**<0.001**

^a^ Core variables for the HIV/AIDS model are: Age, Highest Grade Completed, Food Security, and Religion.

The logistic regression model for a woman’s perceived ability to travel alone to another village for health services (Table 6) indicated that women who had earned money in the last six months had 2.5 times the odds of being able to travel alone when compared to women who had not earned money in the last six months (p < 0.001, [95%CI: 1.73, 3.68]). Further, the odds of being able to travel alone to another village were twice as high for older women compared to women under age 25, and nearly twice as high for women with some schooling compared to those with no schooling. Additionally, women who met the definition for food insecurity had nearly twice the odds of being able to travel for health services compared to women who were food secure (p < 0.05, [95%CI: 1.20, 2.55]). Our final logistic model was created to predict correct knowledge of menstruation (data not shown). After controlling for age, highest grade completed, and food security, women who had heard of HIV/AIDS had 2.4 times the odds of knowing the correct reason for menstruation when compared to women who had not heard of HIV/AIDS (p < 0.001, [95%CI: 1.54, 3.60]).

**Table 6:** Logistic regression model for ability to travel to another village for health services

**Independent variables**	**Unadjusted**			**Adjusted**		
	**OR**	**CI 95%**	***P*-value**	**OR**	**CI 95%**	***P*-value**
Age (ref: <25 years old)						
25-34	1.79	1.12, 2.85	**0.014**	1.99	1.21, 3.29	**0.007**
35+	1.56	0.98, 2.46	**0.058**	2.03	1.23, 3.36	**0.006**
Highest grade completed (ref: no schooling)						
1-3 grade	1.73	1.06, 2.85	**0.027**	1.88	1.13, 3.15	**0.016**
4-6 grade	1.26	0.85, 1.87	0.256	1.66	1.07, 2.58	**0.024**
Food security (ref: food secure)	1.65	1.16, 2.35	**0.006**	1.75	1.20, 2.55	**0.003**
Earned money in the last 6 months (ref: no)	2.56	1.78, 3.70	**<0.001**	2.52	1.73, 3.68	**< 0.001**

Model included 535 respondents; pseudo r^2^ = 6.76%.

## Discussion

This study sought to detect characteristics of positive deviance that occur in the lives of women with low educational attainment, for the purpose of determining whether the sources that influence positive behavior can be enhanced or replicated by groups and organizations that work with women in this region. Using this perspective, our research demonstrates that even modest improvements in formal educational level completed were associated with key outcomes. Further, our results indicate that women earning and saving money expressed higher levels of empowerment, and that women were more likely to receive health education messages related to HIV through media such as television and radio than through friends. These findings are critical in helping practitioners identify focus areas among married women with little or no education where gains might be made to improve health outcomes.

### Delaying marriage and increasing education

Similar to our results, others have demonstrated that age of marriage and educational attainment in girls are associated [48]. Delaying marriage provides girls with the opportunity to pursue further studies. And as our study findings suggest, even modest improvements in formal education have a meaningful impact on women’s health knowledge and behavior. For example, studies have found that formal education is directly correlated with better awareness of HIV/AIDS and reproductive care [1,21,49,50], and have also explored why education, even in small amounts, seems to make a positive impact on health knowledge like HIV/AIDS. Interestingly, in West Bengal, where AIDS awareness is fairly low, the effect of formal education on the odds that an ever-married woman is aware of AIDS has been shown to be greater than the effect of formal education in other Indian states where HIV is thought to be highly prevalent [1]. In this study, compared to women with no formal education, women who had completed class 1–3 were more likely to have heard of HIV/AIDS and be knowledgeable about menstruation, and women who had completed class 4–6 were even more knowledgeable about HIV/AIDS and menstruation.

While the difference between completing classes 1–3 and classes 4–6 might not seem very large and the exact modality of how formal education improves outcomes is not completely understood, these examples demonstrate that small gains in formal education do make a considerable impact on health knowledge and empowerment in adult life. Providing access to primary and secondary education for children has been a continued priority for India [51]. However, for women who are already beyond the age of formal education, this study provides encouraging data for those willing to invest in education. Women with more than 1 year of participation in a SHG had higher odds of correct knowledge of HIV/AIDS compared to women with less than 1 year of participation. Furthermore, increased educational attainment for mothers in this study was also associated with a belief that girls should obtain more education. This confirms recent findings that emphasize the importance of mothers’ education [52], which may also result in better health outcomes for daughters [49]. Given the benefit of even small increases in education, non-formal adult education programs may have a place in improving women’s health as suggested by studies of SHGs, NGOs, and microfinance institutions [17,19,21,25,30]. Additionally, the current study suggests that efforts to keep girls in school for even a few more years may pay large dividends.

### Income and women’s empowerment

The results indicate that women who earned money in the past six months were more likely to report being able to travel alone to seek health care. Similarly, others have shown that in neighboring Bangladesh, income or participation in income-generating activities—typically via microfinance institutions—increase a woman’s ability to move outside the home [53-55]. Consistent with our findings is research from Carr et al. that suggests economic empowerment may be a gateway for improving women’s empowerment [56]. The influence of SHGs, NGOs, and microfinance institutions on the empowerment of mothers may have long-ranging social impacts, such as increasing the number of years of education and delaying the age of marriage for their daughters. The potential for such effects to improve health outcomes is an area for further research.

### Media and health awareness

In this study, women who reported receiving health information from mass media sources had nearly 8 times higher odds of being knowledgeable about HIV/AIDS, compared to women who received health information through social sources. This finding is not unexpected, especially in a country such as India where, in recent decades, mass media has become an important component of multidimensional approaches to educating individuals about HIV/AIDS [57]. Chatterjee reported that most AIDS-related information obtained by women was acquired through mass media channels [58]. Pallikadavath, Sreedharan and Stones note the value of mass media in educating individuals about HIV/AIDS and suggest that in rural India, radio is most likely to be effective [59]. Similarly, and consistent with the results from our study, research has also shown that mass media is an important source of information for reproductive health awareness among adolescent girls [60].

The current study’s findings provide additional validation of India’s investment in mass media reproductive health and HIV/AIDS education. Indeed, the Indian approach may be used as a model of innovation for other developing nations. Within the past decade, the National AIDS Control Organization and the BBC World Service Trust entered into a partnership to produce and disseminate entertainment-education productions through radio and television. The partnership has resulted in widely popular entertainment pieces, which have increased knowledge of HIV/AIDS [57]. Future studies may benefit from investigating the impact on HIV/AIDS information acquired from emerging technologies such as social media. Chatterjee observed that AIDS-related information acquired through mass media may ultimately diffuse into social networks, a process that may be quickened through communication technologies [58].

### Limitations

This study represents a secondary data analysis using available data from a previous study among the same population [31]. Whereas the study was not specifically designed to examine the characteristics of positive deviance among this population, data existed which made possible the current analyses. However, the secondary nature of the data likely limited the ability to test the relative value of many other potentially important factors influencing health outcomes in this population. In addition, the cross-sectional design of the study prevents inference of causal relationships. Further, this study had a sample of women who were 50% Muslim and 50% Hindu, which does not reflect the religious distribution of India, where over 80% are Hindu. However, the Nadia district has a large Muslim minority population that tends to experience more poverty than the Hindu population [61]. Because this is a sample of largely uneducated women in low-income households, it may explain the unique religious distribution in our sample.

Relative to our analysis on a woman’s ability to travel alone to another village for health services, the data did not address availability of health services within their own village or whether other reasons for travel to another village, like employment, were the primary motives to travel alone. Both of these questions offer interesting points of exploration in future studies.

## Conclusions

This was an exploratory analysis of factors associated with positive health outcomes among rural women in West Bengal with low levels of formal education. Study findings indicate that women in disadvantaged circumstances can still achieve good outcomes amidst a host of contextual barriers that would predict poor health outcomes. Positive deviants in this context are shown to be women who are able to earn an income, who have access to information through media sources, and who, even though with little schooling, have marginally higher levels of formal education that lead to improved health outcomes. By knowing these characteristics, organizations working in this area can identify women who fit this description and create action plans to encourage more positive deviant behaviors.

Families, and organizations that support these women, need to ensure girls stay in school as long as it is possible – each year makes a difference. When girls are already outside of formal education, non-formal education for women can fill in knowledge gaps and social support that will lead to positive outcomes. Further, non-formal education needs to emphasize the importance of education and delayed marriage for daughters, as these beliefs are shown to improve a woman’s ability to make better health choices for herself and her family.

Media sources of information matter for improving the lives of rural West Bengali women. Finally, income generating activities for women are important and strategies to increase womens’ access to earning their own income greatly improves their chances of healthier outcomes for themselves and their families.

More research is needed to identify how programs aimed at disadvantaged women can capitalize on any amount of formal education during childhood, as well as how women can increase their access to media in resource poor settings, and how women can engage in income generating activities when a majority of their similarly educated peers are unable to do so.

## Competing interests

Our research represents original, unpublished material. It does not overlap nor duplicate other manuscripts that are under review, in press, or published. We have not submitted this manuscript elsewhere for publication. We have no financial or other contractual agreements that might cause conflicts of interest or be perceived as causing conflicts of interest. There are no company products associated with this research; hence, there are no existing financial arrangement between any of the authors and a company.

## Authors’ contribution

KNGL, LHG, CAR and BTC oversaw the design of the study. KNGL and LHG analyzed the data. All authors interpreted the data, prepared the manuscript, and approved the final version.

## Pre-publication history

The pre-publication history for this paper can be accessed here:

http://www.biomedcentral.com/1471-2458/13/372/prepub
